# Efficacy and Safety of New B Cell-Targeted Biologic Agent for the Treatment of Systemic Lupus Erythematosus: A Systematic Review and Meta-Analysis

**DOI:** 10.3390/jcm12144848

**Published:** 2023-07-23

**Authors:** José L. Gómez-Urquiza, José L. Romero-Bejar, Sara Chami-Peña, Nora Suleiman-Martos, Guillermo A. Cañadas-De la Fuente, Esther Molina, Blanca Riquelme-Gallego

**Affiliations:** 1 Faculty of Health Science, University of Granada, 51005 Ceuta, Spain; jlgurquiza@ugr.es (J.L.G.-U.); briquel@ugr.es (B.R.-G.); 2 Instituto de Investigación Biosanitaria (ibs. GRANADA), 18012 Granada, Spain; jlrbejar@ugr.es; 3 Department of Statistics and Operations Research, University of Granada, 18016 Granada, Spain; 4 Institute of Mathematics, University of Granada (IMAG), 18016 Granada, Spain; 5 Hospital Serranía de Ronda, AGS Serranía de Málaga, Andalusian Health Service, 29400 Ronda, Spain; sara.chami.sspa@juntadeandalucia.es; 6 Faculty of Health Science, University of Granada, 18016 Granada, Spain; norasm@ugr.es (N.S.-M.); gacf@ugr.es (G.A.C.-D.l.F.); 7 Brain, Mind and Behaviour Research Center (CIMCYC), University of Granada, 18016 Granada, Spain; 8 Institute of Neurosciences Federico Olóriz, Biomedical Research Centre, University of Granada, 18012 Granada, Spain

**Keywords:** systemic lupus erythematosus, safety, biologic treatment, efficacy, meta-analysis

## Abstract

Background: B cells are central to the pathogenesis of systemic lupus erythematosus (SLE). We aimed to analyze the efficacy and safety of new B cell-targeted drug therapies for SLE. Methods: A systematic review of randomized controlled trials (RCTs) and reference lists of relevant articles published from inception to 2022 were selected from PubMed, Scopus and Web of Science databases. Random effects meta-analyses were performed to estimate an overall effect size for the risk of adverse events (AEs) and serious adverse events (SAEs) with belimumab and tabalumab treatment. Heterogeneity was assessed using the I^2^ statistic and meta-regression. Funnel asymmetry was evaluated using Egger’s test. Results: This study included 13 RCTs, of which three showed high risk of bias. Egger’s test showed no asymmetry. The risk of SAEs and AEs was lower in the treatment group with belimumab treatment. The risk of AEs for tabalumab treatment was lower in the treatment group and lower for SAEs. Conclusion: Belimumab and tabalumab therapies are effective and safe in the treatment of SLE, although tabalumab does not show sufficient statistical power. Advances in understanding the underlying mechanisms of SLE will be directed towards correlating clinical manifestations with specific pathogenic pathways and the development of precision medicine.

## 1. Introduction

Systemic lupus erythematosus (SLE) is an autoimmune, inflammatory, chronic, multisystemic disease which causes different clinical-biological subgroups since it involves widely differing tissues and organs with diverse clinical symptoms. Furthermore, its incidence and prognosis depend on the age of onset, the sex, the presence of an associated autoimmune disease and the pattern of different autoantibodies [1]. SLE is considered a rare disease; in the EPI-SER2016 study, it was estimated that nine out of ten thousand inhabitants in Spain suffer from lupus, with a 9:1 ratio of women to men [2]. According to the Spanish Academy of Dermatology and Venereology (AEDV), the survival rate of patients with SLE is 90% 20 years after diagnosis [3,4].

There is a high heterogeneity of the genotype and clinical presentation of SLE. Recent studies are encouraging research into the efficacy of more specific drugs with better safety profiles to address the needs of patients who do not respond to conventional treatments, who have developed tolerance, or for SLE subgroups for which there are few current treatments.

B cell activating factor is a critical target for the development of bispecific antibodies. This is a therapeutic goal under investigation that presents great challenges due to the heterogeneity of autoimmune diseases. Other studies have analyzed the effectiveness of therapies targeting type I interferon, cytokines, complement, interleukin-2 and T-cell co-stimulation [4,5]. To date, the only biologic therapy approved for such purposes is belimumab, as although Rituximab is a widely known drug for other diseases, it does not yet have an approved indication for SLE. B cells are central to the pathogenesis of SLE. The production of autoantibodies by autoreactive B cells reacting to autoantigens triggers an overwhelming inflammatory response. Current pathways target surface antigens (CD20-CD22) and growth and survival factors such as B-lymphocyte stimulator (BLYS or BAFF) and proliferation-inducing ligand (APRIL).

In order to provide an update on therapeutic advances targeting B cells for the treatment of SLE, a systematic review and meta-analysis of randomized clinical trials (RCTs) was carried out to analyze the clinical effectiveness and safety of these therapies.

## 2. Materials and Methods

A systematic review of the scientific evidence available to evaluate the effectiveness of new drug therapies targeting B cells has been carried out following the basis of the PRISMA (Preferred Reporting Items for Systematic reviews and Meta Analyses) statement [5].

### 2.1. Search Strategy and Selection Process

The search was performed in Ovid Medline, the Web of Science: Science Citation Index, Cochrane CENTRAL and Scopus and in reference lists of known relevant articles. The following search terms were used: LUPUS AND (BIOLOGICAL TREATMENT OR “ABETIMUS” OR “ATACICEPT” OR “BELIMUMAB” OR “BLISIBIMOD” OR “TABALUMAB” OR “RITUXIMAB” OR “OCRELIZUMAB” OR “EPRATUZUMAB”).

RCTs analyzing the effectiveness of B cell-targeted biologic therapy for the treatment of SLE in the adult population were selected. To collect the most up-to-date evidence, studies published in 2016–2022 were chosen. No language restrictions were implemented. Observational studies, pre-clinical studies, drugs that had not reached phase III for lupus, open-label studies, and case-specific studies were excluded. Trials focused on specific populations (pediatrics, geriatrics) were also excluded. Literature reviews, systematic reviews and meta-analyses were also excluded.

### 2.2. Risk of Bias Assessment

The risk of bias within the trials was assessed separately by two reviewers (EEM and BRG) using the second version of the Cochrane Risk of Bias tool for randomized trials (RoB 2) [6]. The RoB 2 tool encompasses five domains of bias: selection bias, performance bias, detection bias, attrition bias, and information bias. Within each domain, different questions (“signaling questions”) aim to elicit information about trial characteristics that are relevant to the risk of bias. An algorithm based on the answers to the signaling questions proposes a judgment about the risk of bias derived from each domain. The judgments are: “Low” or “High” risk of bias, or may express “Some concerns”.

### 2.3. Data Synthesis

Six meta-analyses were performed calculating the effect size (relative risk) for the risk of adverse events and serious adverse events with belimumab and tabalumab treatment (every 2 weeks or every 4 weeks). I^2^ was used for heterogeneity analysis, Egger’s test for publication bias, and sensitivity analysis was also performed. Meta-analysis was performed for fixed effects if the I^2^ was less than 50% and for random effects if it was greater. Review Manager 5.4 software was used.

## 3. Results

**Figure 1:**
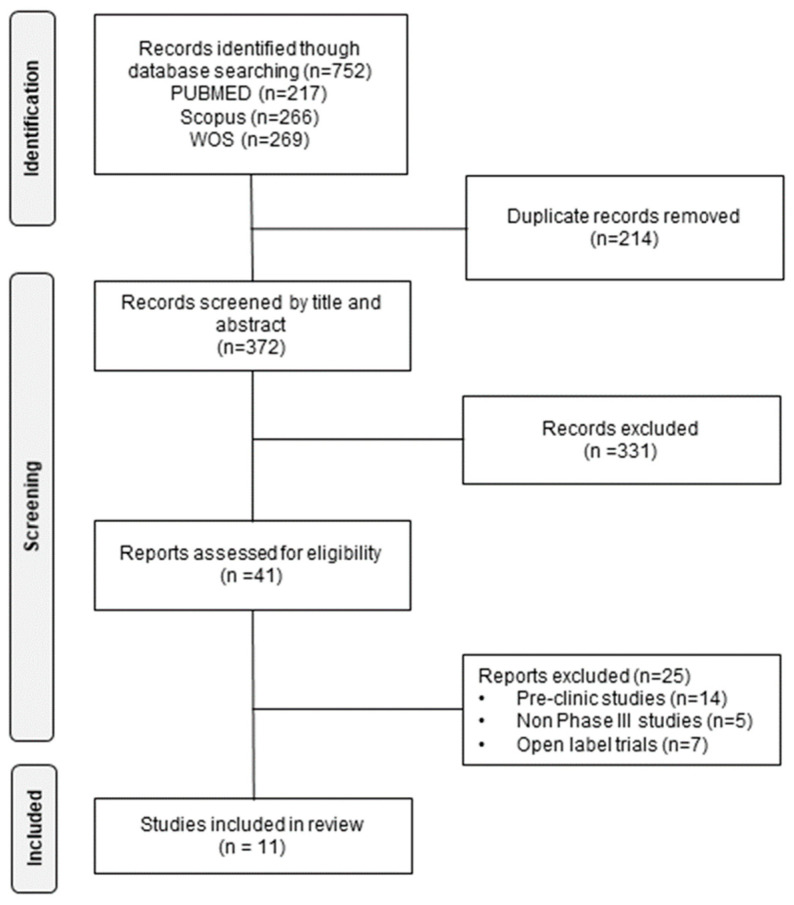
Identification of trials for the systematic review of the effectiveness and safety of B cell-targeted therapies for the treatment of systemic lupus erythematosus.

From the three databases, 752 articles were identified, of which 214 duplicate items were eliminated. After a review by title and abstract, 372 articles were discarded as they did not meet the inclusion criteria. Of the remaining articles, 41 were potentially relevant and were evaluated in detail. Finally, 11 articles were chosen for review (Figure 1).

The characteristics and quality of the 13 RCTs included in the systematic review are presented in Table 1. The effectiveness of seven drugs was analyzed in 8774 patients with different subtypes of SLE. Primary endpoints differed between trials, including: British Isles Lupus Assessment group (BILAG) scale [6,7,8,9], SLE Response Index (SRI) [6,7,9,10,11,12,13,14,15,16], Systematic Lupus Erythematosus Disease Activity Index (SLEDAI (SELENA, 2K)) [9,10,12,14,15,16], steroid sparing and renal response. A summary of B cell-targeted biologic therapies in SLE is shown in Table 2.

### 3.1. Efficacy and Safety of B-Cell Targeted Therapies for the Treatment of Systemic Lupus Erythematosus

#### 3.1.1. BAFF/APRIL Inhibitors

Belimumab is the only biologic agent approved for SLE. The trials included 3125 patients randomized and stratified by SELENA-SLEDAI, complement level and ethnicity (BLISS-SC) and according to induction regimen and ethnicity (BLISS-LN) [6,7,10,12,17,18]. The BLISS-LN trial demonstrated a primary efficacy renal response (43% vs. 32%, OR = 1.6 (95% CI 1.1–2.7); *p* = 0.03) and a complete renal response (30% vs. 20%, OR = 1.7 (95% CI 1.0–2.3); *p* = 0.02) [17]. Regarding the effectiveness of belimumab in the post hoc analysis of the BLISS-LN trial, it was found that belimumab was able to improve the primary efficacy renal response and complete renal response in patients with renal response and complete renal response in patients with proliferative lupus nephritis and/or and baseline creatinine levels below 3 g/g [18]. Significant improvements in SRI4 were observed in both BLISS-SC (OR = 1.68 (95% CI 1.25–2.25); *p* = 0.006) and BEL113750 (OR = 1.99 (95% CI: 1.40–2.82); *p* < 0.0001) at 52 weeks of treatment [6,7]. However, in the EMBRACE study, the response rate to SRI-SLEDAI-2K at week 52 did not improve (belimumab 48.7%, placebo 41.6%; OR = 1.40 (95% CI 0.93–2.11); *p* = 0.107) [10]. However, an improvement in the group compared to placebo was observed in patients who had elevated disease activity or renal manifestations at baseline week. Regarding time to onset of a severe flare, patients treated with belimumab had a significantly lower risk of a kidney-related event or death in BLISS-LN [17] and 50% lower odds of experiencing a severe flare in the BLISS-SC and BEL113750 trials [6,7]. Regarding the EMBRACE study, the safety profile of belimumab correlated with that observed in previous trials [10]. Adverse events (AEs) were the main reason for dropouts in the double-blind phase (belimumab 5.4%; placebo 6.7%). In the BLISS-LN trial the belimumab arm demonstrated greater reductions in DNA-double-stranded and C1q autoantibodies and greater increases in C3 and C4. After normalization of the parameters, these decreased by 58% in belimumab compared to 20% in the placebo group [18].

Atacicept 75 mg and atacicept 150 mg were evaluated in the APRIL-SLE trial [8]. No difference in flare rates was observed between the experimental group treated with atacicept 75 mg and the placebo group (OR 1.15; *p* = 0.543) [8] according to BlyS and APRIL levels. A marked difference in response to atacicept 150 mg was observed in patients with baseline BLyS levels of ≥1.6 mg/mL compared to those with baseline levels of <1.6 mg/mL. In terms of IgG levels, atacicept treatment was associated with reduced rates of flare compared to placebo. In addition, these were the patients who showed the greatest IgG response during the study period. Flare rates were also reduced in patients taking atacicept, with the greatest reductions from baseline in IgM levels (43.6%) and IgA levels (35.9%), as well as in those with the greatest reductions in virgin B cells and plasma cells (42.1% and 47.4%, respectively).

Blisibimod efficacy was analyzed in the CHABLIS-SC1, which included 442 patients who were randomized and stratified by ethnicity, baseline SELENA-SLEDAI score, and proteinuria [13]. The study did not meet the primary efficacy endpoint, SRI (46.9% blisibimod vs. 42.3% placebo; *p* = 0.352), although it was associated with successful steroid tapering (*p* = 0.019) and greater creatinine ratio (UPCR) reductions (*p* = 0.013) in all three proteinuria ranges. In a secondary analysis of SRI-6 response with oral corticosteroids, efficacy was significant, so imbalances in corticosteroid reduction may have influenced primary SRI-6 levels. Blisibimod obtained responses on different biomarkers: non-statistically significant decrease in anti-dsDNA, significant increases in complement C3 and C4, decrease in B cells and significant decreases in mean serum IgM, IgG and IgA immunoglobulin concentrations ranging from 10 to 26%.

Tabalumab has been evaluated in two recent Phase III studies: ILLUMINATE-1 and ILLUMINATE-2 [15,16]. The doses were tabalumab 120 mg every two weeks (Q2W) or every four weeks (Q4W). The trials included 2288 patients (1164 ILLUMINATE-1 and 1124 ILLUMINATE-2) who were randomized and stratified by anti-dsDNA positivity and African ancestry. The ILLUMINATE-1 trial primary endpoint was not achieved in any of the treatment groups’ SRI response (31.8%, 35.2%, 29.3%; *p* > 0.05). In a subgroup of ILLUMINATE-1 patients from a post-hoc trial, a higher response rate was observed in the 120 mg Q2W group than placebo (46.7% vs. 13.3%; *p* = 0.059). In ILLUMINATE-2 the proportion of patients meeting SRI-5 was achieved in the 120 mg Q2W group (38.4% vs. 27.7% in placebo; *p* = 0.002) but not with the less frequent 120 mg Q4W regimen (34.8% vs. 27.7%; *p* = 0.051). Changes in SELENA-SLEDAI scores were also similar in all three groups in ILLUMINATE-1; however, in ILLUMINATE-2 the reduction was greater in both treatment groups. Anti-dsDNA levels decreased significantly in both tabalumab groups versus placebo. In the Q2W dose group, increases in C3 and C4 were observed that were significantly greater than those of placebo. In the 120 Q4W group in ILLUMINATE-1, there was an increase in both C3 and C4, as were serum immunoglobulins in ILLUMINATE-2. In addition, tabalumab was associated with significant increases in mean BAFF concentrations.

#### 3.1.2. CD20/CD22 Inhibitors

In the LUNAR trial (N= 144), changes in BILAG score did not differ between the rituximab and placebo groups (95% CI 1.00–1.56, I^2^ = 0%; *p* = 0.67). Renal response rates were similar in the rituximab and placebo groups (45.8 and 56.9%; *p* = 018) driven by partial response rates (30.6 and 15.30%; *p* = 055). In the population subgroup analysis, a higher response was observed in black patients (rituximab 70% vs. 45% placebo; *p* = 0.20). Reduction in proteinuria, improvement in renal function and need for salvage therapy were shown, but there was no significant difference. Analysis of C4 showed an increase in the rituximab group and a decrease for C3. In peripheral CD19 B cells there was a marked decrease in the rituximab group. However, in the anti-dsDNA there was no significant difference between the two groups.

The BELONG study (N = 378) is the most recent trial to analyze the effectiveness of ocrelizumab. The trial doses were ocrelizumab 400 mg iv or 1g + steroids + mycophenolate mofetil (MMF) or cyclosporine (CYC) 500 mg iv. The mean daily dose of steroids was 6–14 mg and of MMF the target was 3 g/d. The study was terminated early due to more severe infections in patients treated with ocrelizumab. The overall renal response rate (ORR) was higher in the ocrelizumab-treated groups (54.7%, 66.7%, 61.7%) for the placebo, ocrelizumab 400 mg and ocrelizumab 1 g groups, respectively. The mean daily dose of oral prednisone for 48 weeks was not different between the treatment and placebo groups. For both ocrelizumab treatment groups there was a significant increase in complement levels and reduction in anti-dsDNA levels (*p* < 0.001). There was also a more rapid depletion of B cells.

The efficacy and safety of epratuzumab has been studied in EMBODY 1 and EMBODY 2 [9] trials, which included 793 and 791 patients, respectively. Randomization was stratified by geographic region and disease severity (SDI). In terms of efficacy, the primary endpoint was not met in either study, and in improvements in activity there was no significant difference in the proportion of responders between the placebo and epratuzumab groups. Improvements were seen in a multitude of exploratory endpoints and in physician and patient global assessments of disease activity as well as improvements in SF-36 and FACIT-F scores and Lupus QoL scores. However, in all cases, improvements were comparable between the placebo group and the epratuzumab-treated groups. In terms of immune response, a mean reduction of 30–40% in peripheral B cell levels was observed in patients treated with epratuzumab and not in the control group. T-cell, IgA and IgG levels remained stable throughout. IgM levels decreased by approximately 20% from baseline in patients treated with epratuzumab in both studies.

### 3.2. Risk of Bias Assessment

Overall, three (23%) of the included studies were assessed to be at high risk of bias. Seven (54%) studies presented some concerns, and three (23%) studies were at low risk of bias (Table 3). The most common methodological flaw that included the RCTs presented was the lack of adhering to intervention. Additionally, some studies where at high risk of bias due to a lack of statistical analysis plan or had multiple eligible outcome measures, which put them at high risk of reporting bias.

### 3.3. Data Synthesis

Of the studies included in the systematic review, four studies contained data for meta-analysis of belimumab treatment (n = 1530 in the treatment group and n = 879 in the control group) and three studies for tabalumab treatment (n = 768 for the treatment group and n = 770 for the control group). No study was eliminated after sensitivity analysis and there was no publication bias in any of the meta-analyses.

**Figure 2:**
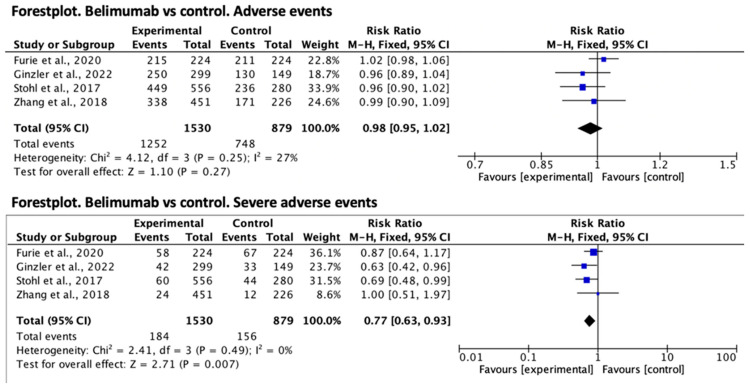
Forest plot for adverse events of belimumab vs. control [6,7,8,9].

With regard to belimumab, the relative risk effect size for the presence of adverse events was RR = 0.98 (95% CI 0.95–1.02) in favor of the treatment group, but was not statistically significant. Regarding serious adverse events, the relative risk was significantly lower in the treatment group, RR = 0.77 (95% CI 0.63–0.93). Both forest plots are shown in Figure 2.

**Figure 3:**
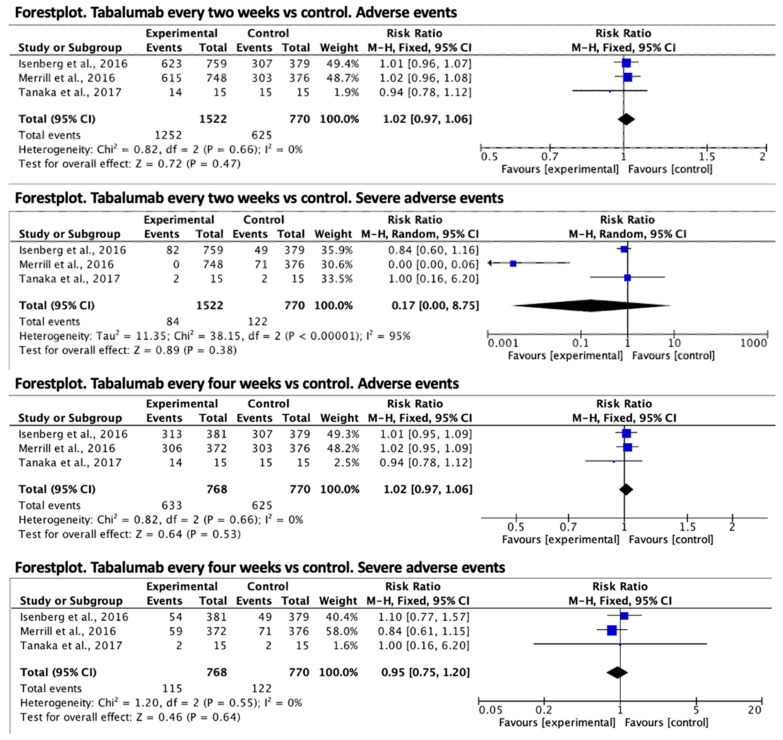
Forest plot for adverse events of tabalumab vs. control [14,15,16].

Regarding tabalumab, in every two-week treatment, the relative risk effect size for the presence of adverse events was RR = 1.02 (95% CI 0.97–1.06) in favor of the control group, but was not statistically significant. Regarding serious adverse events, the result in favor of the treatment group was also not significant, RR = 0.17 (95% CI 0.00–8.75). Finally, in the treatment every four weeks with tabalumab, the relative risk of adverse effects was RR = 1.02 (95% CI 0.97–1.06) in favor of the control group in a non-significant way and for serious adverse effects was RR = 0.95 (95% CI 0.75–1.20) in favor of the treatment group and was non-significant. The forest plots are shown in Figure 3.

## 4. Discussion

The heterogeneity and multisystem involvement of SLE has promoted the development of biologic agents, which, although limited, have made significant progress in recent years. So far, only belimumab has demonstrated an efficacy profile, reducing the number of flares and the need for standard glucocorticoid therapy. Although observational and phase II studies support the use of rituximab and its role in lupus nephritis refractory to standard treatment, the results of phase III RCTs have been disappointing. Does the trial design fail so that drugs effective in phase II are not effective in phase III? Several limitations in trial design have been postulated that may explain the failures.

The major bias in current SLE studies is precisely in the definition of RCTs: the homogeneous sample of patients for trials is a major disadvantage for this pathology. The BLISS-LN study is the only one specific for a clinical manifestation and even so, the information is biased by the various clinical features and pathogenic mechanisms as demonstrated by the failed trial of Davies et al. [20]. On the other hand, the prevalence of severe lupus nephritis and central nervous system disease is underrepresented in all published trials on SLE treatment. Zen, M et al. [21] demonstrated that disease patterns have a clear impact on trial outcome and should be stratified by disease phenotype for patients with disease quiescence, chronically active patients or relapsing-remitting disease. On the other hand, the endpoints and composite indices used may not detect partial improvements, which makes the final analysis and interpretation of the results difficult. This is patent in the Wofsy, D et al. (2013) trial, where the use of different tools led to quite different results [19,22]. Isenberg et al. (2013) [14] suggest that endpoints should be achievable, sensitive and specific, in order not to lose any marginal benefit. In the difficulty to find a more effective outcome measure, the SLEDAI-2K index showed good correlations [23]; however, SRI-50 [24] demonstrated a higher partial response rate, a criterion to be taken into account for future trials.

Another aspect to be taken into account is the standard therapy administered along with the drugs, since it can mask the therapeutic effect. It is difficult to obtain results when the doses of corticosteroids are so varied between groups and do not follow a tapering protocol, so it is necessary to adjust the doses and balance them to minimize bias and be able to compare trials. In addition, the high response rate in the placebo groups has made it difficult to measure the real efficacy of the new drugs; it is still unknown if the withdrawal of the base treatment in these groups would increase the effect of the treatment. What has been demonstrated is that continuous monitoring and regular attention to the patients make them experience greater improvements in the activity of their disease [25].

Anti-dsDNA antibodies and low complement are associated with increased BAFF signaling [21,23,26] and could be used to stratify patients who are more likely to respond to treatment. The results of the present study are in line with Isenberg et al. [14] and the tabalumab trials [15,16], which demonstrate that a marked reduction in anti-dsDNA prevents disease flares, but where a threshold effect occurs where parameters only respond to the highest and most frequent dose of the drug.

The BLISS-BELIEVE study, currently in progress, represents a qualitative advance in the understanding of SLE because, in the absence of a drug targeting more than one pathway, the combination of several drugs may have great therapeutic potential. Since the various clinical manifestations of SLE share non-overlapping immunological pathways [27], more studies should be conducted on this basis.

Finally, with respect to the safety profile, similarities have been found between the treatment and placebo groups in GSD for all the drugs under review; even rituximab showed a lower incidence in the treatment group compared to the control group. A cautious and responsible attitude has led to the suspension of studies such as APRIL-SLE because it could not be determined whether the deaths were caused by the drug. The post hoc analysis of atacicept trials showed that the minimum and maximum drug levels in the deceased patients were relatively low. The most frequent AEs were infections. No anti-drug antibodies were found as shown in the belimumab studies. Only the combination ocrelizumab + mycophenolate mofetil (MMF) was associated with more serious AEs.

The quality assessment of the studies included in this review has reported low results. The RCTs, evaluated with RoB, have shown that the low methodological quality is due to the lack of decisive information to provide veracity to the results. This makes it difficult to obtain real conclusions that can be applied to clinical practice.

Our review has certain limitations. First, we included studies with different outcome measures, inclusion criteria, concomitant treatment and study duration, which makes direct comparison between trials difficult. In addition, other sources of search in databases, gray literature and doctoral theses were not included. Nevertheless, the use of quality assessment tools such as RoB2 provides methodological quality to this review.

## 5. Conclusions

Belimumab and tabalumab therapies are effective and safe in the treatment of SLE, although tabalumab does not show sufficient statistical power. Understanding why these studies have been unsuccessful is critical to design better standardized trial methods and to assess whether further studies are needed to investigate a given drug. Progress in understanding the underlying mechanisms of SLE will be aimed at correlating clinical manifestations with specific pathogenic pathways and, therefore, directed at the development of precision medicine.

## Figures and Tables

**Table 1:** Characteristics of trials included in the systematic review of the effectiveness and safety of B cell-targeted therapies for the treatment of systemic lupus erythematosus.

Study, N	Experimental Treatment (Dose)	Standard Medication	Mean Age	Sex (% Women)	Ethnicity	Follow-Up (Weeks)	Measurement	Inclusion Criteria	Exclusion Criteria	Safety (%)	RoB
Zhang et al., 2018. (BEL113750) N = 677 (226 PBO, 451 belimumab) [7]	Belimumab 10 mg/kg + SoC	Steroids, antimalarials, immunosuppressants, Chinese medicine	PBO: 31.7, EG: 32.3	92.90%	Chinese: 76.2%; Korean: 14.8%; Japanese: 9%	52	SRI4 and 7, ≥4 SS, PGA, BILAG, flares over time	SLE-SS ≥ 8, +ANA, usual therapy	Nephritis, CNS lupus, biologic therapy	AE: PBO: 75.7, EG: 74.9; SAE: PBO: 5.5, EG: 5.3	Some concerns
Stohl et al., 2017. (BLISS-SC) N = 836 (280 PBO, 556 belimumab) [6]	Belimumab 200 mg + SoC	Corticosteroids, antimalarials, immunosuppressive	PBO: 39.6, EG: 38.1	94.70%	Hispanic/Latino: 28.7/71.3%	52	SRI4-8, ≥ 4 SS, PGA, BILAG, time to severe flare and ↓ corticosteroids	SLE-SS ≥ 8, ANA and/or anti-DNA	Severe nephritis or SLE of SNC	AE: PBO: 84.3, EG: 80.8; SAE: PBO: 15.7, EG: 10.8	Some concerns
Furie et al., 2020. (BLISS-LN) N = 448 (224 PBO, 224 belimumab) [17]	Belimumab 10 mg/kg + SoC	Glucocorticoids and immunosuppressants	PBO: 33.1, EG: 33.7	88.00%	Asian: 50%; White: 33%; Black: 14%; American Indian/Alaska Native: 2%; Other: 1%	104	Renal response of primary, complete, ordinal efficacy and time to AE	SLE, ANA, lupus nephritis	Dialysis in 1-year, biological therapy and failures SoC	AE: PBO: 94, EG: 96; SAE: PBO: 30, EG: 26	Low
Ginzler et al., 2022. (EMBRACE) N = 448 (149 PBO, 299 belimumab) [10]	Belimumab 10 mg/kg + SoC	NC	PBO: 39.3, EG: 38.6	96.90%	Black	52	SRI–SLEDAI-2K at week 52	SLE, ANA and/or anti-ADN, SELENA-SLEDAI ≥ 8	Nephritis, SLE of the SNC, EG previous with belimumab	AE: PBO: 87.3, EG: 83.7; SAE: PBO: 22.4, EG: 13.9	Low (double-blind phase, week 52)
Rovin BH et al., 2022. (BLISS-LN) N = 446 (223 PBO, 223 belimumab) [18]	Belimumab 10 mg/kg + SoC	Glucocorticoids and immunosuppressives; cyclophosphamide/azathioprine or mycophenolate mofetil	PBO: 33.1, EG: 33.7	88.10%	Asian: 50%; White: 33%; Black: 14%; American Indian/Alaska Native: 2%; Other: 1%	104	Renal response of primary, complete, ordinal efficacy and time to AE	SLE, ANA, lupus nephritis	Dialysis in 1-year, biological therapy and SoC failures, pregnancy, CNS SLE, active infection 60 days prior, alcohol and/or drug abuse, HIV, Hep B or C	NC	Some concerns
Struemper et al., 2022. (BEL112233) N = 268 (91 PBO, 177 belimumab) [11]	Belimumab 1 mg/kg + SoC	Steroids 4.5%, immunosuppressants 16.4%, antimalarials 43.3%	42.8	93.30%	White: 69.4%; Black or African American: 21.3%; Other: 9.4	312	B cell subset count; serum IgG levels; SRI- 4 response rates; SLE subset plasma and short-lived plasma B cell counts	SLE-SS ≥ 8, +ANA, usual therapy	Acute or chronic disease; AE in BLISS-76	Five serious or severe infections (0.3 per 100 patients/year)	High
Maslen et al., 2021. (BLISS-52, BLISS-76, BLISS-SC) N = 660 [12]	Belimumab 1 mg/kg or Belimumab 10 mg/kg	Prednisone, NSAIDs, antimalarial or immunosuppressive	PBO: 35.1, EG: 35.98	PBO: 95.8, EG: 96.70	Black African ancestry, PBO: 8.55%, EG: 8%	52	SRI4 response rate	SLE, ANA (titer ≥ 1:80), anti-dsDNA antibodies, SELENA-SLEDAI ≥ 6	Severe nephritis, CNS lupus and pregnancy	Severe flare (%): 200 mg subcutaneous: HDA1 (PBO: 28.2, EG 16.1); HDA2 (PBO: 22.7, EG 14.2); Intravenous 10 mg/kg: HDA1 (PBO: 33.0, EG: 20.2); HDA2 (PBO: 30.0, EG: 19.5)	Some concerns
Gordon et al., 2017. (APRIL-SLE) Atacicept. N = 461 [8]	Atacicept 75 mg + SoC or Atacicept 150 mg + SoC	Corticosteroids	NC	NC	NC	52	BILAG outbreak, time to first severe outbreak and safety	Positive SLE in ANA and anti-ADN, ≥1 BILAG	Patients with glomerulonephritis or SLE of the CNS	NC	High
Merrill et al., 2018. (CHABLIS SC-1) Blisibimod. N = 442 (197 PBO, 245 blisibimod) [13]	Blisibimod 200 mg Q1W + SoC	Corticosteroids, immunosuppressive, antimalarial, MTX	PBO: 35.6, EG: 36.7	93.00%	Black: 3.2%; White: 67.8%; Asian: 28.8%; Native Pacific Islander: 0.2%	52	SRI4 and 6, UPCR, time to first severe flare and ↓ corticosteroids	ANA or anti-dsDNA, 4 ACR criteria, SS ≥10	Active vasculitis, SLE of the NS or nephritis, recent biological therapies	AE: PBO: 64.8, EG: 67.6; SAE: PBO: 7.1, EG: 6.6	Low
Isenberg et al., 2016. (ILLUMINATE-1) Tabalumab. N = 1164 [14]	Tabalumab 120 mg Q2W + SoC or Tabalumab 120 mg Q4W + SoC	NSAIDs, corticosteroids, antimalarials, and immunosuppressants	PBO: 39, EG Q2W: 40, EG Q4W: 40	93.30%	White: 55.1%; Hispanic/Latino: 31.3%; Asian: 17.1%; American Indian: 16.4%; Black: 10.5%; Other: 0.6%	52	SRI5, time to first severe flare, ↓ corticosteroids, change in brief fatigue inventory (BFI)	SLE ≥ 4 ACR criteria, SLEDAI-2K ≥ 6 +ANA and/or anti-dsDNA	Nephritis, SLE of the NS, use of biological therapies	AE: PBO: 81.1, EG Q2W: 82.1, EG Q4W: 82.3; SAE: PBO: 12.9, EG Q2W: 11.1, EG Q4W: 14.4	Some concerns
Tanaka et al., 2017 (ILLUMINATE-1 subgroups) Tabalumab. N = 45 [15]	Tabalumab 120 mg Q2W + SoC or Tabalumab 120 mg Q4W + SoC	NSAIDs, corticosteroids, antimalarials, and immunosuppressants	PBO: 41, EG Q2W: 36, EG Q4W: 41	97.70%	Japanese: 100%	52	SRI5, ↓ corticosteroids, SLEDAI2K, time to first severe flare, PGA, BFI	ILLUMINATE-1 subgroup		AE: PBO: 100, EG Q2W: 93.3, EG Q4W: 93.3; SAE: PBO: 13.3, EG Q2W: 0 EG, Q4W: 13.3	Some concerns
Merrill et al., 2016 (ILLUMINATE-2) Tabalumab. N = 1124 (376 PBO, 748 tabalumab Q2W, 372 tabalumab Q4W) [16]	Tabalumab 120 mg Q2W + SoC or Tabalumab 120 mg Q4W + SoC	NSAIDs, corticosteroids, antimalarials, and immunosuppressants	PBO: 42, EG Q2W: 41, EG Q4W: 42	92.20%	White: 65.9% Hispanic/Latino: 26.8%; Black: 12.5%; Asian: 9.9%; American Indian: 8.3%; Other: 3%; Native Pacific Islander: 0.26%	52	SRI5, time to first severe flare, corticosteroid reduction, change in brief fatigue inventory	SLE ≥ 4 ACR criteria, SLEDAI-2K ≥ 6 +ANA and/or anti-dsDNA	Nephritis, SLE of the NS, conventional therapy dose change, biological therapies	AE: PBO: 80.6, EG Q2W: 82.2, EG Q4W: 82.4; SAE PBO: 18.9, EG Q2W: 12.4, EG Q4W: 16	Some concerns
Clowse et al., 2017. (EMBODY 1-2) Epratuzumab. N = 1529 (512 PBO, 1017 epratuzumab) [9]	Epratuzumab 600 mg + SoC or Epratuzumab 1200 mg + SoC	Corticosteroids, immunosuppressants (MMF, methotrexate, leflunomide, and azathioprine), and antimalarials	EMBODY I PBO: 41.2, EG: 42.2; EMBODY II PBO: 41.1, EG: 41	EMBODY I 93.20%; EMBODY II 93.50%	EMBODY I and II: Hispanic: 19.7–19.8%; White: 74.6–75.5%; Black: 12.2–11.1%; Asian: 8.9–3.3%	48	SRI4, SLEDAI, BICLA, BILAG, corticosteroid dose change	SLE ≥ 4 ACR criteria, BILAG-2004 A n ≥ 1, B n ≥ 2, SLEDAI-2K ≥ 6 and +ANA and/or anti-dsDNA	Nephritis or serious neuropsychiatric SLE	AE: PBO: 85, EG 600: 83.5, EG 1200: 86.1; SAE: EMB. PBO: 17.7, EG 600: 17.1, EG 1200: 17	High

ACR: American College of Rheumatology criteria, ANA: antinuclear antibodies, BILAG: British Isles Lupus Assessment Group index, CNS: symptoms of central nervous system, EG: experimental group, Hep B: hepatitis B, Hep C: hepatitis C, HIV: human immunodeficiency virus), IgG: immunoglobulin G, MTX: methotrexate, NC: not computed, NSAIDs: non-steroidal anti-inflammatory drugs, PBO: placebo group, PGA: Physician Global Assessment, Q2W: every 2 weeks, Q4W: every 4 weeks RoB: risk of bias, SLE: systemic lupus erythematosus, SLEDAI: SLE disease activity index, SLE-SS: SLE with Sjögren’s syndrome, SoC: standard of care, SRI4: the Systemic Lupus Erythematosus Responder Index-4, UPCR: urine protein-to-creatinine ratio.

**Table 2:** Characteristics and mechanisms of action of treatments used in the trials included in the systematic review.

Target	Treatment	Mechanism of Action	Characteristics	Clinical Trials
BlyS/APRIL	Belimumab	Blocks BLyS	Reduces the differentiation of B lymphocytes into plasma cells that produce immunoglobulins	Approved by FDA BLISS-LN [17,18] BLISS-SC [6] BLISS-52 [12] BLISS-76 [12] BEL112233 [11] BEL113750 [7]
	Atacicept	Blocks BlyS and APRIL	Acts as a decoy receptor and interferes with the interaction of cytokines with their receptors related to BCMA and BLyS-R	APRIL-SLE [8]
	Blisibimod	Blocks BlyS soluble and membrane-bound	Presents little toxicity; has more affinity for BLyS than belimumab	CHABLIS-SC1 [13]
	Tabalumab	Blocks BlyS soluble and membrane-bound	Neutralizes BLyS but does not bind APRIL	ILLUMINATE 1-2 [15,16]
CD20	Rituximab	Depletes B cells by complement-dependent cytotoxicity, antibody-dependent cytotoxicity and activation of apoptosis	Immunogenicity occurs due to the chimeric nature	LUNAR [19]
	Ocrelizumab		Higher binding affinity; improved ADCC and less CDC compared to rituximab	BELONG
CD22	Epratuzumab	CD22 phosphorylation and decreased BCR signaling; less vigorous B cell depletion	Non-depleting immunomodulatory agent; cannot induce complement-dependent cellular cytotoxicity	EMBODY 1-2 [9]

ADCC: antibody-dependent cell-mediated cytotoxicity, APRIL: a proliferation-inducing ligand, BCMA: B cell maturation antigen, BLISS-LN: Belimumab International Study in Lupus Nephritis, BLISS-SC: subcutaneous belimumab, BlyS: B lymphocyte stimulator, CD: cluster of differentiation, CDC: complement-mediated cytotoxicity, FDA: Food and Drug Administration.

**Table 3:** Risk of bias assessment of individual studies included in the systematic review of the effectiveness and safety of B cell-targeted therapies for the treatment of systemic lupus erythematosus [6,7,8,9,10,11,12,13,14,15,16,17,18].

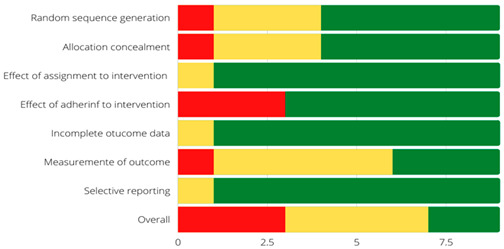													
	Zhang, 2018 [7]	Stohl, 2017 [6]	Furie, 2020 [8]	Ginzler, 2022 [9]	Rovin BH, 2022 [10]	Struemper H, 2022 [11]	Maslen T 2022 [12]	Gordon 2017 [13]	Merri ll 2018 [14]	Isenberg 2016 [15]	Tanaka 2017 [16]	Merrill 2016 [17]	Clowse 2017 [18]
Random sequence generation	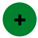	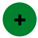	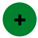	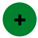	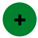	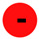	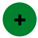	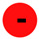	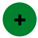	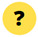	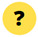	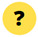	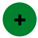
Allocation concealment	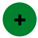	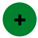	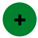	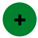	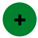	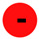	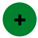	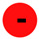	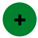	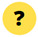	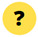	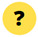	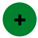
Effect of assignment to intervention	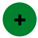	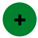	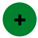	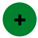	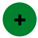	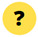	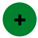	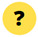	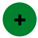	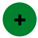	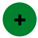	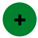	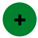
Effect of adhering to intervention	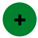	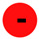	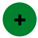	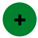	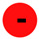	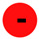	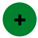	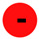	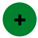	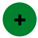	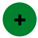	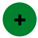	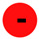
Incomplet outcome data	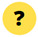	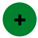	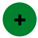	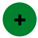	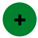	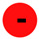	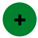	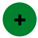	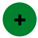	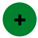	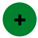	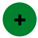	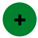
Measurement of outcome	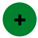	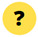	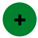	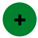	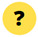	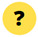	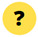	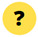	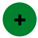	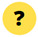	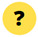	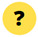	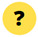
Selective reporting	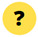	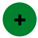	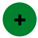	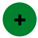	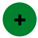	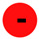	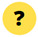	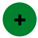	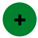	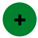	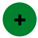	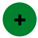	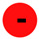
Overall	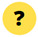	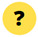	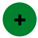	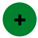	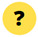	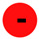	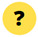	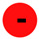	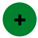	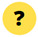	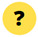	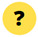	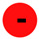
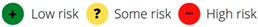													

## Data Availability

Not applicable.
